# Genome-Wide Association Study Identifies a Novel Canine Glaucoma Locus

**DOI:** 10.1371/journal.pone.0070903

**Published:** 2013-08-07

**Authors:** Saija J. Ahonen, Elina Pietilä, Cathryn S. Mellersh, Katriina Tiira, Liz Hansen, Gary S. Johnson, Hannes Lohi

**Affiliations:** 1 Department of Veterinary Biosciences and Research Programs Unit, Molecular Neurology, University of Helsinki, Helsinki, Finland; 2 The Folkhälsan Institute of Genetics, Helsinki, Finland; 3 Department of Equine and Small Animal Medicine, Faculty of Veterinary Medicine, University of Helsinki, Helsinki, Finland; 4 Canine Genetics Animal Health Trust, Lanwades Park, Kentford, Newmarket, Suffolk, United Kingdom; 5 Department of Veterinary Pathobiology, College of Veterinary Medicine, University of Missouri, Missouri, United States of America; University of Rochester, United States of America

## Abstract

Glaucoma is an optic neuropathy and one of the leading causes of blindness. Its hereditary forms are classified into primary closed-angle (PCAG), primary open-angle (POAG) and primary congenital glaucoma (PCG). Although many loci have been mapped in human, only a few genes have been identified that are associated with the development of glaucoma and the genetic basis of the disease remains poorly understood. Glaucoma has also been described in many dog breeds, including Dandie Dinmont Terriers (DDT) in which it is a late-onset (>7 years) disease. We designed clinical and genetic studies to better define the clinical features of glaucoma in the DDT and to identify the genetic cause. Clinical diagnosis was based on ophthalmic examinations of the affected dogs and 18 additionally investigated unaffected DDTs. We collected DNA from over 400 DTTs and a genome wide association study was performed in a cohort of 23 affected and 23 controls, followed by a fine mapping, a replication study and candidate gene sequencing. The clinical study suggested that ocular abnormalities including abnormal iridocorneal angles and pectinate ligament dysplasia are common (50% and 72%, respectively) in the breed and the disease resembles human PCAG. The genetic study identified a novel 9.5 Mb locus on canine chromosome 8 including the 1.6 Mb best associated region (p = 1.63×10^−10^, OR = 32 for homozygosity). Mutation screening in five candidate genes did not reveal any causative variants. This study indicates that although ocular abnormalities are common in DDTs, the genetic risk for glaucoma is conferred by a novel locus on CFA8. The canine locus shares synteny to a region in human chromosome 14q, which harbors several loci associated with POAG and PCG. Our study reveals a new locus for canine glaucoma and ongoing molecular studies will likely help to understand the genetic etiology of the disease.

## Introduction

Glaucoma belongs to a heterogeneous group of hereditary optic neuropathies and is one of the most common causes of irreversible blindness [1], [2] characterized by a progressive degeneration of the retinal ganglion cells (RGC) and optic nerve [3]–[5]. Elevated intraocular pressure (IOP) is considered a strong risk factor, which results from an obstruction to the normal flow of aqueous humor flow through the anterior chamber and the trabecular meshwork [6]. Aqueous humor is secreted posterior to the iris by the ciliary body and it flows anteriorly to the anterior chamber.

Glaucoma can be a primary or secondary disease. Hereditary glaucoma is broadly classified into primary open-angle (POAG), primary closed-angle (PCAG) and primary congenital glaucoma (PCG) [7] where the IOP may or may not be elevated [8]. Primary glaucoma can be classified on the basis of the filtration angle as well, where the iridocorneal angle (ICA) may be open, narrow or closed or the ciliary cleft may be open, narrow or collapsed. POAG is the most common form of human glaucoma with open iridocorneal angles and possible elevation of IOP [8]. PCAG is a result of elevated IOP and the subsequent death of photoreceptor cells. Elevated IOP is caused by a shallow anterior chamber and the obstruction of the iris-trabecular meshwork in the iridocorneal angle of the eye, which causes blockage of the aqueous humor outflow [9]. PCG is the most common form of childhood glaucoma and it is characterized by abnormalities in the anterior chamber angle and elevated IOP. Ocular abnormalities in PCG cause obstructed aqueous humor outflow. Onset of PCG usually occurs within the first few years of life [10].

In human the genetic basis of glaucoma has not been completely established due to its heterogeneous etiology resulting from the interaction of multiple genes and environmental factors [7], [11], [12]. To date, four genes, *myocilin (MYOC)* [13], *neurotrohin 4 (NTF4)* [14], *optineurin (OPTN)* [15] and *WD repeat domain 36 (WDR36)* [16] and at least 20 genetic loci of which 14 are named (GLC1A-N) [17], [18] have been associated with human POAG. Three loci (GLC3A-3C) [18] and two genes *cytochrome P450 1B1 (CYP1B1)* [19] and *latent transforming growth factor beta binding protein 2 (LTBP2)* [20] have been associated with PCG, which is an autosomal recessive developmental disorder. Recently three loci have been associated with PCAG, in which separate markers showed significant association after replication on human chromosomes 1, 8 and 11 [9]. However, no causative genes have been associated with PCAG. Furthermore, multiple genes and loci have been associated with syndromes and other ocular conditions accompanied by glaucoma [18].

In addition to humans, open and closed-angle glaucomas have been described in several dog breeds [21]. Clinical features resemble largely human glaucoma including loss of the RGCs and an elevated IOP. Abnormalities in the pectinate ligament (PL) structure are also considered as a risk factor [22]–[24]. Pectinate ligament form the internal boundary of the canine iridocorneal angle. In the normal canine eye the pectinate ligament is presented as pillar of tissue, which project from the base of iris to the peripheral Descemet’s membrane and provides support for the iris to the posterior cornea [25].Although structural abnormalities are considered a risk for the disease, not all dogs affected with pectinate ligament dysplasia develop glaucoma.

Despite existence of the disease in many breeds the genetic etiology of glaucoma is almost completely unknown in dogs. So far a mutation in only one gene, *ADAM metallopeptidase with thrombospondin type 1 motif*, 10 (*ADAMTS10*), has been identified as a cause of recessive POAG in a research colony of Beagles [26].

To better understand the molecular background of the disease we have embarked on a clinical and genetic study of primary glaucoma in Dandie Dinmont Terriers (DDT). The breed is affected with a slowly progressing disease, which resembles clinically human PCAG and affects the middle-aged and elderly dogs before 10 years of age. However, little is known about the disease etiology in the breed. Several abnormalities associated with the drainage or iridocorneal angle are detected in glaucoma affected dogs prior to the elevation of IOP. In addition, pectinate ligament dysplasia (PLD) is considered a significant risk factor for glaucoma in the breed according to the eye examination reports by veterinary ophthalmologists.

Our study describes the clinical features in the affected and unaffected DDT populations and successfully establishes a study cohort to map a novel canine glaucoma locus on CFA8.

## Materials and Methods

### Clinical Study

A complete ophthalmic examination was performed on 18 Finnish Dandie Dinmont Terriers that had not been diagnosed with glaucoma, whose ages ranged from 3–13 years, including basic neuro-ophthalmic examination, tonometry, indirect ophthalmoscopy, slit lamp biomicroscopy and gonioscopy.

After neuro-ophthalmic examination (menace response, dazzle reflex and pupillary light reflexes), a topical anesthesic (oxybuprocaine; Oftan Obucain, Santen, Tampere, Finland) was instilled and the intraocular pressure was measured using an applanation tonometer (Tonopen XL, Medtronic Solan, Jacksonville, FL, USA). Gonioscopy was then performed using either an 18 or 19 mm Koeppe goniolens (Ocular Instruments, Bellevue, Washington, USA) with carbomer gel (Viscotears 2 mg/g, Alcon, Vantaa, Finland) as coupling media. With the goniolens positioned on the cornea, a handheld slit lamp (Kowa SL-15, Kowa Ltd, Japan) with a 10-fold magnification was used to visualize the entire iridocorneal angle (ICA). The width of ICA was studied based on method described by Ekesten *et al*. [27] and Bjerkås [22]. Pupils were then dilated with topical 0.5% tropicamide (Oftan Tropicamid) and slit lamp biomicroscopy and indirect ophthalmoscopy (Welch-Allyn 12500 BIO, Skaneateles Falls,NY,USA) were performed to examine the anterior and posterior segments of the eye.

The anterior width of the ciliary cleft, the distance between the origin and the insertion of the pectinate ligament, and the total distance from the origin of the pectinate ligament to the anterior corneal surface were subjectively evaluated. The ratio between these variables was estimated subjectively and used as an estimate of the relative width of the opening of the ciliary cleft (RWOCC). The RWOCC was graded as open, slightly narrow, narrow or closed. PL was considered to be normal if less than 50% of it was affected by PLD.

The degree of the pectinate ligament dysplasia was categorized based on the European College of Veterinary Ophthalmology (ECVO, http://www.ecvo.org) guidelines in both eyes. The ICA was judged to be affected by PLD when it exhibited abnormally broad and thickened pectinate ligament fibres or solid sheets of pectinate ligament tissue, with or without “flow holes” over 25% or more of the circumference. Dogs with PLD were graded 0–3 based on the percentage of dysplasia present [28], 0 representing normal pectinate ligament and 3 extensive PLD (Table 1). PLD and ICA values were evaluated based on the left eye for practical reasons. Previous studies have reported unilateral reporting to be sufficient as both eyes are generally found to be equally affected [24], [27]. Intra ocular pressure was measured on undilated eyes from all 18 unaffected dogs and post-dilation from 16 dogs. None of the dogs had been previously diagnosed with PLD or glaucoma or had clinical signs of elevated IOP.

**Table 1:** Categorization of PLD severity.

Severity (%) of PLD	Degree of PLD
≤25	0
25–50	1
50–75	2
≥75	3

Categorization of PLD severity based on the percentage of abnormal PL.

Statistical analysis of the clinical data was performed using SPSS statistic package. The association between PLD and ICA narrowing and age was calculated using Spearman correlation coefficient. Possible PLD or narrowing of ICA and association to gender was evaluated using non-parametric Kruskall-Wallis test due to the small number of dogs (n = 18) clinically examined in the study. The association between PLD and glaucoma was determined using Chi-square test by comparing glaucoma affected dogs with PLD diagnosed prior to the development of glaucoma (n = 9) to dogs with PLD (n = 35) but no glaucoma at the time of examination. The association test included both dogs that were clinically examined in this study and dogs whose ophthalmological reports were received. For the rest of the glaucoma affected dogs, clinical data prior glaucoma development was not available.

### DNA Samples

Blood and buccal swab samples were collected and submitted to the DNA Animal Repository at the University of Missouri, USA and to the canine DNA bank at the University of Helsinki, Finland. All samples were submitted with the owners’ consent and were collected under the permission of animal ethical committee of County Administrative Board of Southern Finland (ESLH-2009-07827/Ym-23). EDTA blood samples were collected from 33 DDTs diagnosed with glaucoma and 159 control dogs from 10 countries including Czech Republic, Denmark, Finland, Germany, Hungary, the Netherlands, Norway, Sweden, the United Kingdom and the United States. In addition, 35 samples were collected from DDTs affected with PLD. All dogs were eye examined by certified veterinary ophthalmologists at least once and were healthy or diagnosed either with an unilateral or bilateral glaucoma or PLD. Control dogs for the GWAS were over 7 years old and confirmed to have healthy eyes by a veterinary ophthalmologist although the presence of mild PLD (grade 1) was tolerated. Fine mapping study included dogs over 5 years of age as well. A pedigree was constructed by GenoPro genealogy software around the affected dogs using the genealogical data available in public canine registries such as the Finnish Kennel Club’s Koiranet or as informed by the owners.

### Genomic DNA Isolation

Genomic DNA was extracted from EDTA blood samples, using Chemagic Magnetic Separation Module I (MSM I) (Chemagen Biopolymer-Technologie AG, Baeswieler, Germany) according to the manufacturer’s instructions. DNA from buccal swabs (Eurotubo Cytobrush, sterile, 200 mm, Danlab) was extracted using QIAamp DNA Mini Kit (Qiagen). DNA concentration was measured using Nanodrop ND-1000 UV/Vis Spectrophotometer (Nanodrop technologies, Wilmington, Delaware, USA) and stored at −20°C.

### Genome Wide Association Study

A genome-wide association study was performed in a cohort of 23 cases and 23 controls using Illumina’s Canine SNP20 BeadChip arrays (San Diego, CA, USA). Genotyping was performed in our core facility at the FIMM Technology Center. Quality control procedures were included when analyzing the data. Only SNPs which confirmed to Hardy-Weinberg expectations P< = 0.0001, had ≥95% genotyping rate and minor allele frequency (MAF) of 5% were included in the analysis, resulting in the exclusion of 8700 SNPs out of 22260. The GWAS data is available upon request.

To compare the allele frequencies, a case-control association test was performed using PLINK 1.07 analysis software [29]. Significance values from this analysis were used to generate a whole-genome association plot using the statistical package R [30]. Identity-by-state (IBS) clustering and CMH meta-analysis (PLINK) were used to adjust for population stratification. Genome-wide corrected empirical p-values were determined by applying 50.000 permutations to the data.

### Fine-mapping and Analysis of Other Breeds

Fine mapping was performed with 190 DDTs including 33 cases and 157 controls and 110 additional SNP markers (∼1 marker/100 kb) between 18 Mb to 29 Mb on CFA8 using iPLEX SEQUENOM MassARRAY platform (San Diego, CA, USA). SNPs and individuals, which had at least 65% genotyping rate and MAF 5%, were included in the analysis resulting in the exclusion of 16 SNPs out of 110 SNPs and 20 individuals out of 190. Association and haplotype analysis for 3-SNP and 5-SNP haplotypes were performed using PLINK sliding window option.

The DDT glaucoma locus was tested in three other breeds affected with glaucoma or PLD including, Flat Coated Retriever (10 cases and 10 controls), Siberian Husky (4 cases and 4 controls) and Welsh Terrier (5 cases and 5 controls) by Sanger sequencing the three best associated SNPs (BICF2P594410 g.19928718 bp, BICF2P377952 g.21323113 bp, BICF2S23025995 g.23018375 bp) (Table S3). Siberian Huskies were diagnosed with uni- or bilateral glaucoma and raised IOP while Flat Coated Retrievers and Welsh Terriers presented mild to severe PLD with narrowed or closed ICA.

PCR primer pairs were designed to amplify the SNPs from genomic DNA from cases and controls in each breed (Table S1). PCRs were carried out in in 12 µl reactions consisting of 0.6 U Biotools polymerase (Biotools, Madrid, Spain), 200 µM dNTPs (Thermo Scientific), 1.5 mM MgCl_2_ (Biotools), 1×PCR buffer (Biotools), 0.83 µl of forward and reverse primers (Sigma Aldrich) and 10 ng template genomic DNA. 5% dimethyl sulfoxide **(**DMSO) and additional MgCl_2_ was added according to supplemental data (Table S2). The reaction mixtures were subjected to a thermal cycling program of 95°C for 10 min, followed by 35 cycles of 95°C for 30 s, 30 s at the annealing temperature, and 72°C for 60 s and final elongation stage of 72°C for 10 min. The PCR products were purified using ExoSap (Thermo Scientific) according to manufacturer’s instructions and sequenced using the Sanger sequencing. Sequence analysis was performed using Variant Reporter software (Applied Biosystems, Foster City, California, USA).

### Candidate Gene Sequencing

The coding sequence and flanking splice sites of candidate genes *CTAGE-family, member 5 (CTAGE5), F-Box protein 33 (FBXO33), leucine rich repeat and fibronectin type III domain containing 5 (LRFN5), pinin (PNN)* and *trafficking protein particle complex 6B (TRAPPC6B)* were PCR amplified using the same protocol as for the SNPs and screened by capillary sequencing. List of primers is shown in supplemental data (Table S2).

## Results

### Study Cohort, Pedigree and Clinical Study

A large multinational study was established with a total of 440 DDTs from 10 countries including 33 cases and 157 controls. All affected dogs in the study were diagnosed either with glaucoma together with elevated IOP (>15 mmHg) and closed ICA, or glaucoma and PLD. No information was available on possible optic nerve defects from the affected dogs. The average age of the glaucoma diagnosis was at 7.7 years, however the actual onset is likely earlier as most of the affected dogs visit the veterinary clinic only after the symptoms have already advanced to a high IOP and impaired ICA and PLD structures. Controls dogs in the study were older, examined by veterinary ophthalmologists and without any signs of glaucoma with elevated IOP.

Based on the clinical data of 33 glaucoma affected dogs, nine dogs presented significantly narrowed or completely closed PL’s and developed glaucoma after the PLD diagnosis, suggesting PLD as a risk factor in DDTs (P = 0.05). For the rest of the 24 glaucoma affected dogs in this study clinical data was not available before the glaucoma diagnosis.

To further investigate the ocular structures in the DDT breed and to define the exclusion or inclusion criteria for the control dogs, we performed comprehensive ophthalmic examinations including basic neuro-ophthalmic examination, tonometry, indirect ophthalmoscopy, slit lamp biomicroscopy and gonioscopy for 18 unaffected Finnish DDTs (7 males and 11 females, between 3 to 13 years). All unaffected dogs had normal IOP, and no difference was observed in IOP before and after dilation. Fundi were normal in 14 of the studied dogs. Four dogs had slight arteriole narrowing on the periphery of the retina, which is probably due to the age of the dogs (8–13 years). However, several other ocular abnormalities were observed in the majority of the studied unaffected dogs. PLD was diagnosed in 13 (72.3%) dogs, including, one with a severe dysplasia (grade 3), nine with grade 2 abnormalities and three with grade 1 changes (Table 2). Abnormal ICA width was found in nine dogs, of which four had a slightly narrow, two narrow and a closed ICAs. In the rest of the dogs with normal ICAs, PL’s were mostly normal with some slight changes in less than 50% of the circumference of the PL (<50%). Four of the studied dogs had no ocular changes with both PLD and ICA grade 0. No association between age or gender and PLD or ICA narrowing was observed (p_PLD_ = 0.45, p_ICA_ = 0.71, p_PLD_ = 0.40, p_ICA_ = 0.77, respectively).

**Table 2:** Degrees of PLD.

Degree of PLD	Number of dogs	% of dogs	Age (years)
0	5	27.8	3–9
1	3	16.7	4–11
2	9	50.0	4–13
3	1	5.6	6
Total	18	100.0	

Number and percentage of dogs with different degrees of PLD.

### Genetic Analyses

The pedigree established around the 33 affected dogs indicates a high breed incidence, which suggests a genetic contribution to glaucoma in the breed. However, the definitive mode of inheritance is difficult to determine due to missing phenotypes and late onset of the disease (Fig. S1).

**Figure 1:**
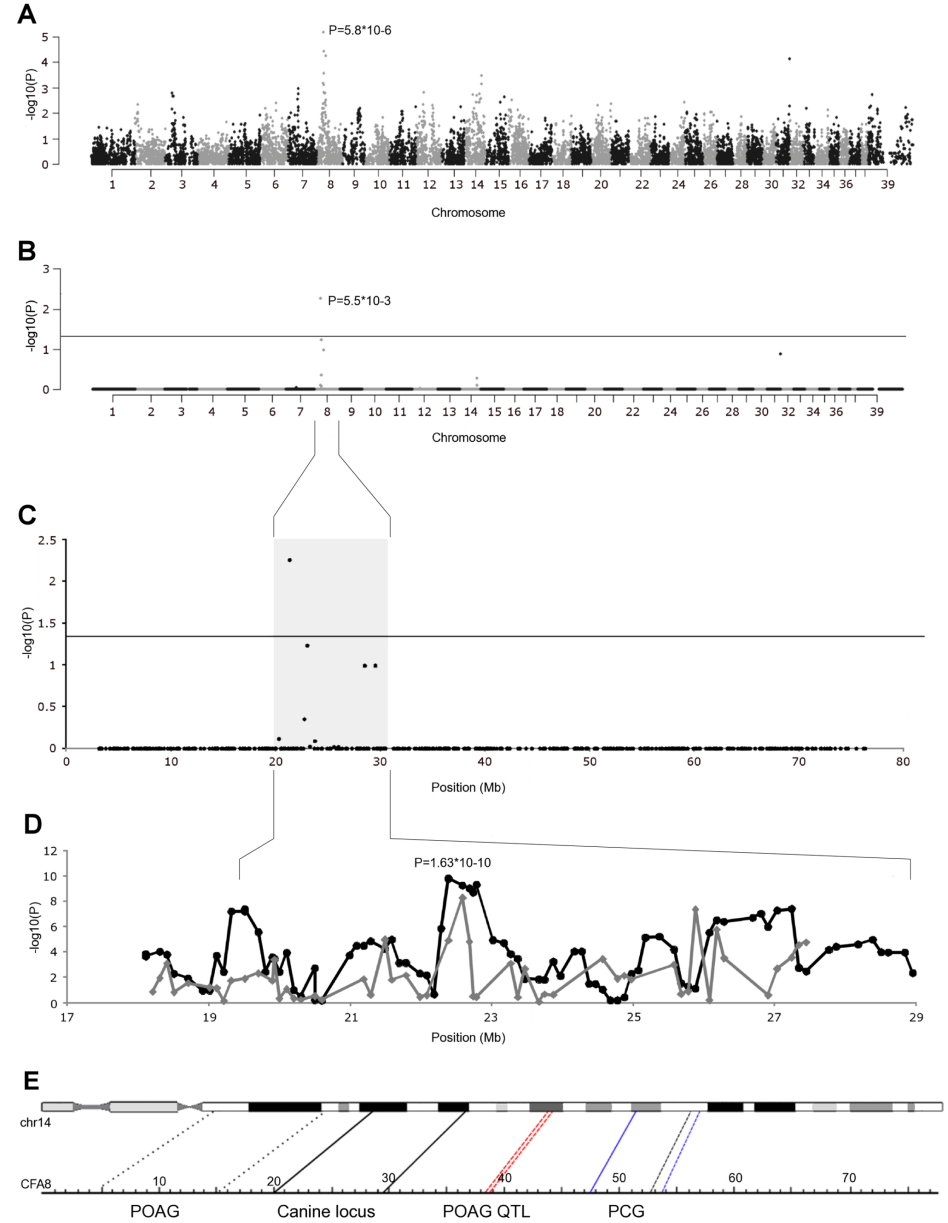
Genome wide association analysis. **A**) A Manhattan plot of genome-wide case-control association analysis performed using 23 cases and 23 controls. **B**) Results of the genome-wide association analysis after 50,000 permutations. A significant association was identified on CFA8 (P = 5.83×10−6, Pgenome = 5.5×10−3). Horizontal line indicate the 5% significance level **C**) The glaucoma associated region on CFA8 spans from 20.0 Mb to 29.5 Mb. **D**) Results of the fine-mapping narrowed the best association to 22.0 and 23.6 Mb (P = 1.63×10−10). Gray line shows the individual SNP association, black line indicates the 5-SNP haplotype. **E**) The canine CFA8 locus (solid black line) maps to a human chromosome 14 to a 11 Mb region between 39.2 Mb to 50.4 Mb. Previously mapped GLC3C locus for PCG between 77.4 Mb to 78.6 Mb, maps to canine CFA8 at a region between 52.8 Mb to 53.9 Mb (dashed black line). Another PCG locus was mapped on chr14 between 71.5 Mb to 78.6 Mb which overlap the GLC3C locus (solid blue line). Two POAG loci were mapped on chr14 between 20.8–33.97 Mb (dotted black line) and a QTL locus between 60.79–61.0 Mb (dashed red line).

A genome-wide association analysis was performed to map the glaucoma locus in a cohort of 23 glaucoma affected and 23 unaffected DDTs. Association analysis revealed a 9.5 Mb associated region with genome wide significance ranging from 20040105 bp to 29518099 bp on CFA8 (build 2.1 of the canine genome reference sequence) the best SNP, BICF2P377952 (p_raw_ = 5.83×10^−06^, p_genome_ = 5.5×10^−3^) (Fig. 1A–C). The genome-wide SNP data was used for IBS clustering, resulting in the identification of 10 clusters and an inflation factor of 1.4 and a presence of slight population stratification. To correct for stratification a CMH meta-analysis was carried out using PLINK. The same association signal on CFA8 was identified (p_raw_ = 2.21×10^−05^, p_genome_ = 0.02).

A fine-mapping was performed to narrow down the critical region with 110 additional SNPs across the region (18–29.5 Mb) in a total cohort of 33 glaucoma-affected DDTs and 157 control dogs. This cohort included 10 additional cases and 134 controls as a replication. This replication cohort identified the strongest SNP BICF2P751027 G>A at g.22737570 bp (p = 6.3×10^−05^). The strongest association for the entire study cohort was found for the SNP BICF2P1308530 T>C at g.22785650 bp for the T allele (p = 5.33×10^−09^, OR = 6.6, 52% frequency in cases and 14% controls). Homozygosity for the T allele further increased the risk to 32-fold (95% CI 3.7–280.8, 19% frequency in cases and 0.7% controls). A 5-SNP haplotype sliding window approach narrowed down a 1.6 Mb best associated region between 22.0 and 23.6 Mb (p = 1.63×10^−10^) (Fig. 1D). Besides the best associated region, three other significantly associated regions at 19.7 (p = 2.77×10^−6^) Mb, 25.57 (p = 6.57×10^−5^) Mb and 26.9 (p = 1.03×10^−6^) Mb were found (Fig. 1D).

The best associated 1.6 Mb region contained 7 protein coding genes of which none have been associated with glaucoma or PLD before. We selected five genes (*CTAGE5, FBXO33, LRFN5, PNN, TRAPPC6B*) for mutation screening based on gene function or position nearest to the most highly associated markers. Exonic sequencing covering also the splice sites in four DDT cases and four controls revealed six coding and 12 intronic variants (Table S4). However, none of the variants were case-specific and are therefore unlikely causative for the disease. This agrees with the bioinformatic prediction of the pathogenicity of the non-synonymous mutations using Polyphen2 [31] and SIFT-programs [32].

Finally, the three most highly associated SNPs (BICF2P594410 g.19928718 bp, BICF2P377952 g.21323113 bp, BICF2S23025995 g.23018375 bp) were genotyped in four other breeds affected with glaucoma or PLD but no association to either phenotype was observed in any of them (Table S3).

## Discussion

This study describes late-onset PCAG in DDTs and identifies its clinical features and genetic locus. The clinical study in unaffected dogs revealed that the breed is commonly affected with significant ocular abnormalities, including PLD and narrowed or closed ICAs. In addition, PLD was present in many affected dogs prior to glaucoma development. However, while most clinically examined dogs had ocular abnormalities with severe PLD and ICA narrowing, they did not develop glaucoma signs even at older age (>10 years). A previous study has reported PLD and narrowing iridocorneal angles to be predisposing to glaucoma development in dogs [25]. Dogs affected with glaucoma have more severe PLD changes compared to normotensive dog with usually very mild PLD and narrowing of the ICA [22]–[24]. Our clinical findings are supported by these studies and suggest that while the structural abnormalities may increase the disease risk in DDTs, additional factors influence the development of glaucoma, including the locus discovered in this study.

Glaucoma is diagnosed at or after middle age (>7 years) in DDTs. The fact that most affected DDTs have already a raised IOP and closed iridocorneal angles at the time they visit the veterinary clinic suggests that the primary symptoms may have gone unnoticed and the actual age of onset may be earlier. This study cannot formally exclude the possibility that the disease is congenital and that the early physiological changes develop very slowly until the observed onset at the middle age. More detailed ophthalmic examinations at different ages are warranted to better define the disease onset, including the ultrasound of iridocorneal angle, examination of the optic cupping and cup-to-disk ratio as well as the recording the ERG responses. The retinas were normal ophthalmoscopically in all clinically examined PLD affected dogs and no photoreceptor death was identified. However, it would be of interest in the future to measure ERG responses which may become abnormal as the disease progresses towards glaucoma.

Based on the previous studies, PLD, the width of the ICA and glaucoma are known to be inherited in dogs [22], [26], [27], [33]. Our GWAS data identified a glaucoma locus with a 6.6-fold allelic risk, which differs from the previously described locus in a colony of Beagles [26]. Homozygosity further increased the glaucoma risk (OR = 32), although the confidence interval was extensive due to small number of cases. Increased OR for homozygosity supports recessive mode of inheritance and likely a reduced penetrance. However, although a single genomic region was discovered, our pedigree includes an example of a litter where both parents are affected but not all the littermates have developed glaucoma. The litter has clearly past the average age of diagnosis (7.7 years) being now almost 12 years. Our fine mapping data across the entire locus indicated 1.6 Mb best associated region, however, the region includes at least three other strongly associated loci and it is possible that this locus contains more than one affected gene. A larger sample size would have to be collected to identify possible additional risk loci in other chromosomes.

The associated region (9.5 Mb) includes 21 genes, however, none of them have been previously associated with glaucoma in any species. Of the five selected genes, only one of them, *PNN* has been previously associated with an ocular phenotype, modulating the activities of developmental factors of anterior eye segments, lens and cornea, respectively [34]. Exonic sequencing did not reveal causative variants in any of the selected genes, and we have initiated a targeted re-sequencing of the entire associated region in selected cases and controls to identify the causative gene.

DDT glaucoma locus on CFA8 shares synteny to a region in human chromosome 14q21.1-q21.3 (39.2–50.4 Mb) (Fig. 1E). Chromosome 14 has been associated several times in human PCGs and POAG but not PCAG. The GLC3C locus on chr14q24.3 (76.3–82.1 Mb) has been associated with human PCG in one multigenerational, consanguineous Pakistani family [35]. Chen *et al*. narrowed the locus further between 77.4–78.6 Mb by studying PCG in Chinese Han population [36]. Firasat *et al.* found another PCG locus overlapping the GLC3C locus on chr14q24.2–24.3 between 71.5–78.6 Mb [37]. Wiggs *et al.* identified a locus for POAG between markers D14S261 and D14S121 (20.8–33.97 Mb, respectively) using linkage analysis in sibpairs [38]. Another risk locus for POAG was identified at 61.0 Mb between *SIX homeobox 1* (*SIX1*) and *SIX homeobox 6* (*SIX6*) genes by studying optic nerve area and vertical cup-to-disc ratio [39]. The same marker was mapped by Osman *et al.* in Japanese population with POAG where the association extended to 60.79 Mb [45]. None of the human PCG or POAG loci at chr14 directly overlap our canine locus.

The clinical phenotype in DDTs resembles mostly human PCAG including shallow or closed anterior chamber and the obstruction of the iris-trabecular meshwork contact in the iridocorneal angle of the eye, which further causes blockage of the aqueous humor outflow and elevated IOP. Several candidate genes related to PCAG in different human populations have been identified. Vithana *et al.* mapped three susceptibility loci for PCAG, in chromosomes 1, 8 and 11 [9]. *Matrix metalloproteinase-9* (*MMP-9*), *membrane type frizzled related protein* (*MFRP*), *methyleneterahydrofolate reductase* (*MTHFR*), *heat shock protein 70* (*HSP70*) and *retinal homeobox* (*CHX10*) genes have been suggested as candidate gene for PCAG as well [40]–[43]. However, the findings have not been replicated in other populations and are somewhat controversial [44]. Although canine PCAG resembles human PCAG, more detailed clinical comparisons would be warranted to better define similarities between the species. However, none of the suggested human PCAG loci overlap the DDT locus, and it is possible that our novel glaucoma locus suggests also a novel locus for human PCAG at chr14q within the cluster of glaucoma loci.

In conclusion, we have discovered a novel glaucoma locus in DDTs, whose clinical features resemble human primary closed-angle glaucoma. Given that the associated locus does not include any known genes, our ongoing targeted re-sequencing studies are likely to reveal novel glaucoma genes for both dog and human. Our study revealed significantly increased glaucoma-risk in homozygous dogs. Although a genetic risk test could be developed to screen homozygous dogs in the breeding programs, the identification of the actual causative variant would be more informative and efficient tool as a genetic test given the small gene pool of the DDT breed.

## Supporting Information

Figure S1 Pedigree of glaucoma affected Dandie Dinmont Terriers. The pedigree suggested that glaucoma in DDTs is inherited but the exact mode of inheritance is difficult to determine due to missing or unconfirmed phenotypes. The pedigree includes litters where both parents are affected. However, not all the littermates become affected suggesting a single locus with reduced penetrance or more complex mode of inheritance.
(TIF)

Table S1 List of SNPs and primers used for replication of the association on CFA8 in other breeds.
(XLSX)

Table S2 Primers used for sequencing of the coding regions and flanking splice sites of candidate genes.
(XLSX)

Table S3 The best associated SNPs were genotyped in four other breeds affected with glaucoma or PLD but no association to risk allele (A1) was identified in any of them.
(XLSX)

Table S4 The results of the sequencing of the candidate genes identified 6 coding and 12 intronic variants. None of the coding variants were expected to be causative.
(XLSX)
