# Supplementary figures and images for: Genome-Wide Association Study Identifies a Novel Canine Glaucoma Locus
Source: PLoS One. 2013 Aug 7;8(8):e70903. doi: 10.1371/journal.pone.0070903 (PMC3737263; doi:10.1371/journal.pone.0070903)

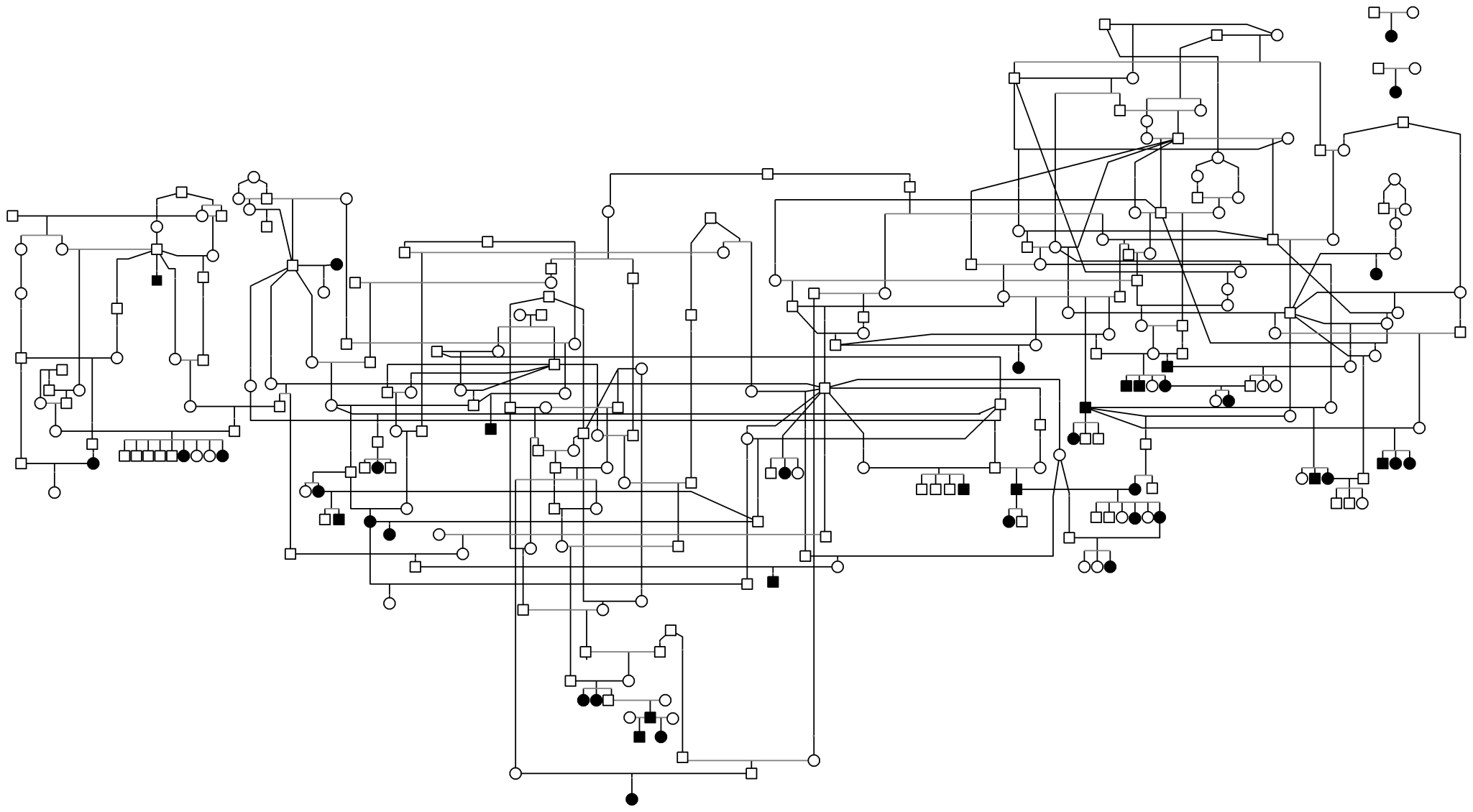

Supplement: Figure S1 — Pedigree of glaucoma affected Dandie Dinmont Terriers. The pedigree suggested that glaucoma in DDTs is inherited but the exact mode of inheritance is difficult to determine due to missing or unconfirmed phenotypes. The pedigree includes litters where both parents are affected. However, not all the littermates become affected suggesting a single locus with reduced penetrance or more complex mode of inheritance. (TIF) [file pone.0070903.s001.tif]
